# Error in Byline

**DOI:** 10.1001/jamanetworkopen.2026.33455

**Published:** 2026-08-14

In the Invited Commentary titled “Magnetic Resonance Imaging–Based Staging in Prostate Cancer—Are We Ready?”^1^ published July 15, 2026, there was an error in Chadi Nabhan’s degrees in the byline. This article has been corrected.^1^
